# Trends in gastrointestinal cancer burden in Zimbabwe: 10-year retrospective study 2009–2018

**DOI:** 10.3332/ecancer.2025.1839

**Published:** 2025-02-06

**Authors:** Tinashe Adrian Mazhindu, Ntokozo Ndlovu, Margaret Borok, Vincent Aketch Nyangwara, Pageneck Chikondowa, Marie Hidjo Madeleine, Collen Masimirembwa, Onesai Chihaka, Edith Matsikidze, Charley Jang, Kevin Grimes

**Affiliations:** 1 African Institute of Biomedical Science and Technology, Harare, Zimbabwe; 2 Department of Oncology, Medical Physics and Imaging Sciences, Faculty of Medicine and Health Sciences, University of Zimbabwe, Harare, Zimbabwe; 3 Department of Chemical and Systems Biology, Stanford University School of Medicine, Stanford, CA 94305, USA; 4 Department of Medicine, Faculty of Medicine and Health Sciences, University of Zimbabwe, Harare, Zimbabwe; 5 Sydney Brenner Institute for Molecular Bioscience, Faculty of Health Sciences, University of the Witwatersrand, The Mount, 9 Jubilee Road, Parktown 2193, Johannesburg, Gauteng, South Africa; 6 Department of Human Genetics, Faculty of Health Sciences, University of the Witwatersrand, Johannesburg 2000, South Africa; 7 Surgical Gastroenterology Clinic, Harare, Zimbabwe; 8 Department of Medicine, NYU Langone Health, New York, NY 10016, USA

**Keywords:** gastrointestinal cancer, Zimbabwe, Africa, non-communicable diseases

## Abstract

**Background:**

As one of the non-communicable diseases, cancer will overtake communicable, maternal, neonatal and nutritional diseases combined as the leading cause of mortality by 2040. Gastrointestinal (GI) cancers are predicted to increase by over 50% in the next 20 years, with a higher incidence in developing countries. In this study, we describe the national GI cancer trends in Zimbabwe using the annual reports from the Zimbabwe National Cancer Registry (ZNCR) from 2009 to 2018.

**Methods:**

Demographic data and incidence of GI cancer subtypes were collected and analysed from the ZNCR annual reports from 2009 to 2018. Age standardised rates (ASRs) for each GI cancer subtype were calculated and simple trend analysis was performed over the 10-year study period.

**Results:**

In total, 10,859 new GI cancer cases were reported during the study period, accounting for 17.2% of all cancers in Zimbabwe and 55% of these were males. The most prevalent GI cancers were oesophageal, liver, gastric, colon and rectal malignancies. In males, on average the incidence of ASR of oesophageal, liver and gastric cancer increased annually by 14.7%, 17% and 16%, respectively. In females, on average the ASR of oesophageal, liver and gastric cancer increased annually by 27.2%; 18% and 13%, respectively. Overall, one in ten new cases of oesophageal cancer were diagnosed in patients under 45 years of age and for liver cancer, one in four new male cases were diagnosed below the age of 45 years.

**Conclusion:**

Zimbabwe faces an increasing trend in all GI cancer subtype incidence over the decade reviewed. The rate of increase in oesophageal and gastric cancers in females was particularly high and the male-to-female ratio observed requires further etiological studies. The increasing rate of young GI cancer patients requires both education regarding risk factors and national screening policies that are tailored to the Zimbabwean population's characteristics and context.

## Background

Globally, gastrointestinal (GI) malignancies account for 1 in 4 new cancer cases (approximately 4.8 million) and are responsible for 1 in 3 cancer deaths (approximately 3.4 million) annually [1]. The most common GI cancers globally in order of incidence are gastric, hepatic, oesophageal, pancreatic and colorectal [1]. Most cancers in Africa are diagnosed at an advanced stage resulting in a poor prognosis [2–5]. Contributing factors include a lack of nationwide screening programs; inaccessibility to essential services in pathology, imaging, surgery, radiotherapy and other oncology, particularly in rural areas; and a larger emphasis of healthcare systems on infectious diseases, leaving non-communicable diseases (NCDs) including cancer under resourced [6]. This resource allocation gap may worsen given projections that NCDs in Africa will overtake communicable, maternal, neonatal and nutritional diseases combined as the leading cause of mortality in Africa between 2030 and 2040 [7, 8].

GI cancers are predicted to increase by over 50% in the next 20 years, with a higher incidence in developing countries [9]. Contributing factors for this increase in Africa include population growth, increased lifespan and changes in lifestyle and diet. Common modifiable risk factors for GI cancers include cigarette smoking, obesity, excessive alcohol intake, sedentary lifestyle, high consumption of red and processed meats, and low consumption of fruits, vegetables, dietary fibre and dietary calcium [10–13]. Infections with *Helicobacter pylori*, human papilloma virus, hepatitis virus B and C and Human Immunodeficiency Virus (HIV) also contribute to specific cancer types. The pattern of these risk factors varies between Africa and other parts of the world and within Africa itself [14]. Changes in lifestyle, including dietary modifications shifting from traditional foods to less-healthy Western diets, including refined carbohydrates, high-fat foods and processed red meat, have been observed [15, 16]. Obesity, which used to be rare in Africa, has been increasing particularly in urban dwellers who are more likely to adopt unhealthy diets and lifestyles [17]. Genetic risk factors are also reported to be more important in Africa given the proportion of patients diagnosed below the age of 40 years. In Zimbabwe among patients with colorectal cancer, 1 in 18 have a genetic predisposition [18, 19].

Local variations in modifiable risk factors, the prevalence of cancer-associated infections and health services will impact each country’s cancer incidence trends overtime [20]. Reviewing changes in GI cancer burden over time illuminates local etiological patterns and assists in estimating future incidence and required resources for cancer management. Historical trend analysis helps both policy and clinical planners prioritise, optimise and implement public health preventative interventions for GI cancers. GI cancers, unlike the topically dominant cervical cancer, require extensive multimodality diagnostic capacity including endoscopic, open surgical and/or minimally invasive image-guided biopsies. The risk factors in an individual or community along with the diagnostic capacity of the health delivery system contribute to the cancer trends in a country. Previously reported colon and gastric cancer trends analysis for Sub-Saharan Africa have reported average annual percentage changes that ranged from 0.54 to as high as 2.8 [21, 22]. Additionally, early onset gastric cancer has been reported to be increasing in both males and females in Sub-Saharan Africa.

In this study, we describe the national GI cancer trends in Zimbabwe over a 10-year period from 2009 to 2018 using data from the Zimbabwe National Cancer Registry (ZNCR) annual reports [19, 20]. We also examine the evolution of the cancer registry over the 10-year period and the need for continued improvement in cancer data availability for Zimbabwe to inform national policies and programs aimed at improving cancer prevention and treatment.

## Materials and methods

### Study setting

In Zimbabwe, cancer is a growing major cause of morbidity and mortality. Access to screening, early detection, diagnostic and palliative care services is limited due to resource constraints and are centralised, so limiting access for many patients. The complement of cancer diagnostics, staging and treatment services in the public health system is currently only available at two state central referral hospitals in the two largest cities, Harare and Bulawayo. This places an access, time and cost limitation to accessing diagnostics in a population where only 10% of people have health insurance [25]. Most cancer patients in Zimbabwe are diagnosed with locally advanced and metastatic disease [4, 5, 26]. Primary prevention with vaccinations for HBV and HPV are now well established; however, no screening or early detection programmes exist for GI cancers in the country [27].

### Data collection

The ZNCR is a population-based cancer registry founded in 1985 and led by a collaborative effort between the Zimbabwe Ministry of Health and Child Care and the International Agency for Research on Cancer of the World Health Organisation. ZNCR is a multi-source national cancer registration and surveillance system that utilises active and passive methodologies to record cases. Cancer case notification forms are filled out for cancer cases incident at central and regional public hospitals, private hospitals, all public and private pathology laboratories, radiotherapy centres and palliative and hospice care centres. The national death registry also provides vital status support to the ZNCR. Cancer notification forms capture basic demographic, diagnostic, disease features and basic treatment. The registry utilises the 10th edition of the International Statistical Classification of Diseases and Related Health Problems (ICD-10) to classify patients. Incidence, cancer site, age and sex characteristics of malignant neoplasms of digestive organs, classified C15- C26 according to the International Classification of Diseases 10th Revision codes (ICD-10), were extracted exclusively from ZNCR annual reports from 2009 to 2018 [28]. Incidence and demographic data were extracted from the ZNCR annual reports from 2009 to 2018 for oesophageal, gastric, small intestine, colorectal, rectal, anal, liver, gallbladder and pancreatic malignancies. When available, limited mortality data were also extracted and analysed.

### Statistical analysis

A crude incidence rate was defined as the number of new cancers of a specific site occurring annually per 100,000 population. The Zimbabwe census population count for 2012 was used and exponential growth based on this census was used to estimate the population for the period reviewed. The annual population growth rate was reported as 1.5% [29]. The age-adjusted rate which is a statistical measurement that controls for age differences was calculated as a weighted average of the age-specific (crude) rates. The weights were the proportions of persons in the corresponding age groups of the population. Age-adjusted rates were derived for each GI cancer subtype. To produce age-standardised rates (ASRs) we multiplied each age-specific (crude) rate by the appropriate weight and summed the products. The 10-year percent changes and average annual incidence increase in ASR were calculated using the formula:


$$\mathrm{Trend}\;\mathrm{Percentage}\;=\;\frac{(\mathrm{Current}\;\mathrm{Period}\;\mathrm{Value}\;-\;\mathrm{Base}\;\mathrm{Period}\;\mathrm{Value})}{(\mathrm{Base}\;\mathrm{Period}\;\mathrm{Value})}\;\times \;100\;[30]$$

Statistical formula tables for all calculations are outlined in Supplementary statistical sheet.

## Results

### Cancers incidence and mortality cases over 10 years 2009–2018

**Figure 1.**
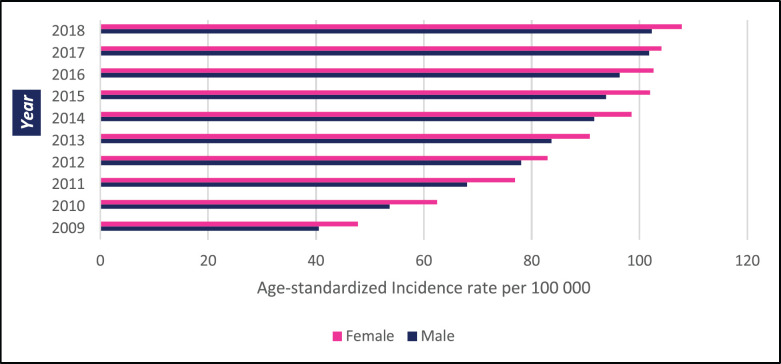
10-year ASR for all cancers in Zimbabwe 2009–2018.

**Figure 2.**
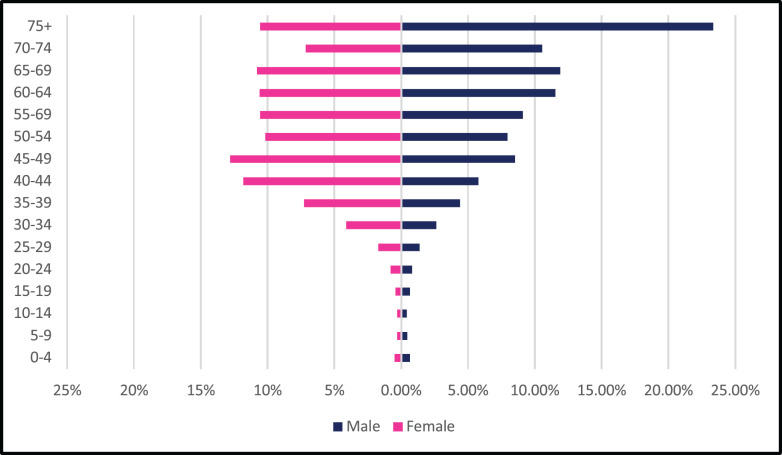
ASR per 100,000, females, males- all cancer 2009–2018.

In Zimbabwe, 63,195 new cases of cancer were diagnosed between 2009 and 2018 with 26,677 male and 36,518 female cases giving a male-to-female ratio of 1:1.4. From 2009 to 2018 the annual total of new cancer cases more than doubled from 3,519 to 7,841. The annual age-standardised incidence rate (ASR) over the 10-year period increased consistently throughout the period as shown in Figure 1. In males, the annual ASR for all new cancer cases increased from 40.5 per 100,000 in 2009 to 102 per 100,000 in 2018. In females, the annual ASR for all new cancer cases increased from 47.8 per 100,000 in 2009 to 109.8 per 100,000 in 2018. The ASR increased over the 10-year period by 152.3% in males and 125.6% in females. The average annual increase in ASR for all cancer types was higher in males than females (11.3% versus 9.87%). Analysing ASR by age sub-grouping showed that 27.3% and 17.1% of all new cancer cases were diagnosed before the age of 45 years in females and males, respectively (Figure 2). Between 2009 and 2018; 21,238 cancer-related deaths were reported in Zimbabwe with females accounting for 53% of cases and a male-to-female ratio of 1:1.1. Overall crude mortality rate increased from 8.4 per 100,000 to 18.4 per 100,000. Male overall crude mortality increased by 117% from 8 per 100,000 to 17.4 per 100,000 from 2009 to 2018 while female crude mortality rate increased by 119% from 8.8 per 100,000 to 19.3 per 100,000.

### GI cancer incidence over 10 years 2009–2018

In Zimbabwe, 10,859 new GI cancer cases were diagnosed between 2009 and 2018 with males accounting for 55% of cases and a female-to-male ratio of 1:1.2. Over this 10-year period, annual GI cancer cases increased by 131%. GI cancers accounted for 17.2% of all incident cancer cases in Zimbabwe. In the 10-year period, male GI cancer crude incidence rate increased from 5.7 per 100,000 to 11 per 100,000 and female increased from 3.4 per 100,000 to 8 per 100,000 translating to an increase of 93% and 135%, respectively. Detailed mortality data available for each GI cancer was only available in the 2017 and 2018 ZNCR reports. In 2017 and 2018, the age-standardised mortality rate (ASMR) for all GI cancers in females was 11.4 per 100,000 and 11.7 per 100,000, respectively. For males, ASMR for all GI cancers for 2017 and 2018 was 13.5 per 100,000 and 12.7 per 100,000, respectively. The leading cause of death among GI cancers in females was esophageal followed by gastric and liver malignancies while in males it was esophageal followed by liver and gastric malignancies.

### Esophageal cancer incidence over 10 years 2009–2018

**Figure 3.**
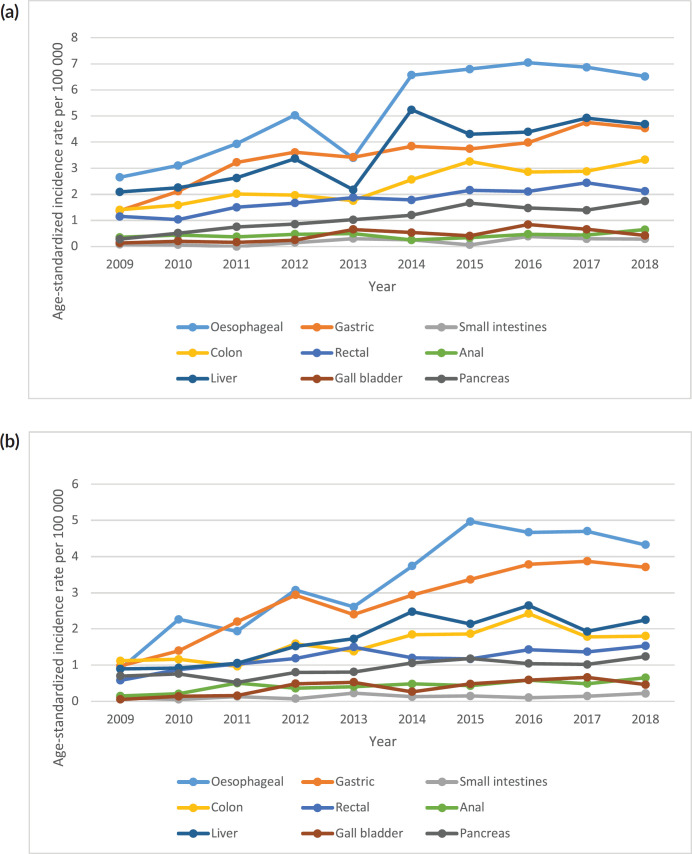
(a): All GI cancers in males by subtype ASR for Zimbabwe 2009–2018. (b): GI cancer in females by subtype ASR for Zimbabwe 2009–2018.

**Figure 4.**
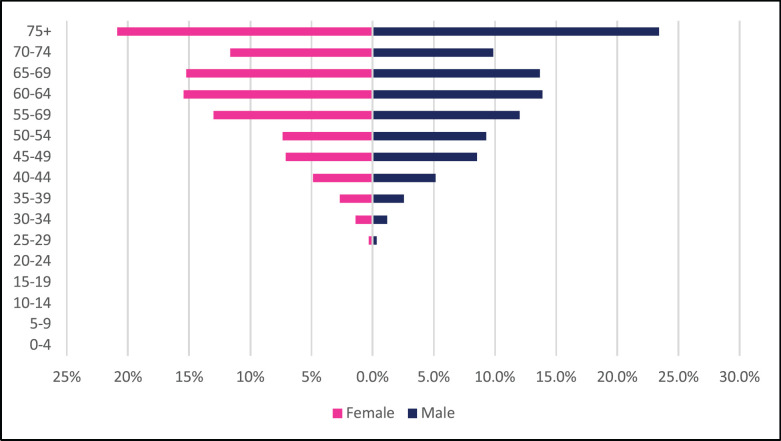
Esophageal cancer ASR per 100,000 females and males from 2009 to 2018.

**Supplementary Figure 1.**
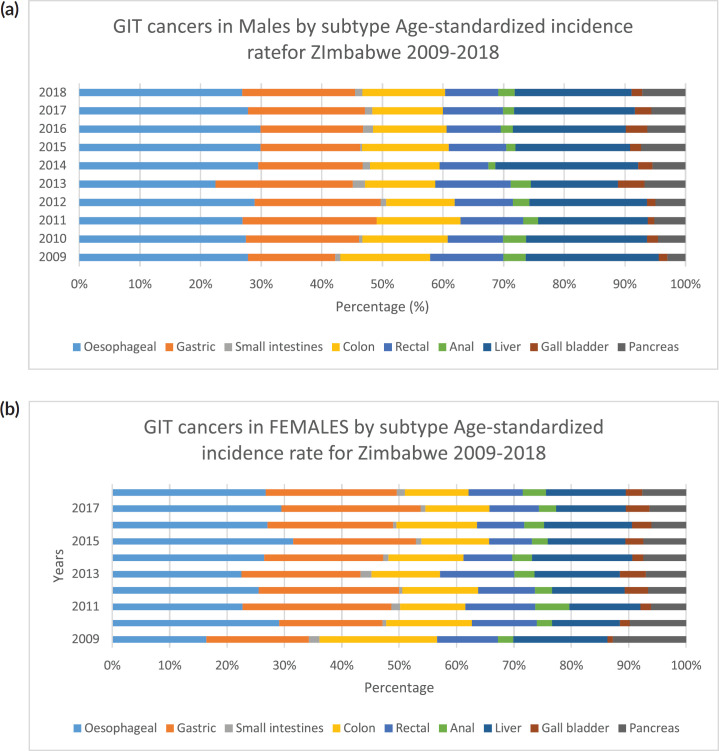
(a): GIT cancers in males by subtype ASR for Zimbabwe 2009–2018. (b): GIT cancers in Females by subtype ASR for Zimbabwe 2009–2018.

A total of 2,984 incident esophageal cancer cases were diagnosed between 2009 and 2018 with males accounting for 58% of cases. Esophageal cancers accounted for 4.72% of all incident cancer cases in the 10-year period. Among males, esophageal cancer accounted for 6.49% of all incident cancer cases while in females it accounted for 3.43% of all cancers. Esophageal cancer represented 27.5% of all GI incident cancer cases making it the most common GI cancer in Zimbabwe (Supplementary Figure 1a and b). Over the 10-year period, the ASR for esophageal cancer increased in males by 146% from 2.7 per 100,000 to 6.5 per 100,000 and in females increased by 382% from 0.8 per 100,000 to 6.5 per 100,000 with an incidence ratio of 1:1 in 2018. Furthermore, the average annual increase in ASR was 14.7% in males and almost double this was observed in females at 27.2%. The ratio of female to male cases decreased from 1: 3.4 in 2009 to 1:1 in 2018 (Figure 3a and b). In an age sub-population analysis, a similar trend was observed with both males and females having approximately 1:5 incident cases diagnosed in patients above 75 years of age and 75% of all cases were diagnosed between the ages of 40–74 years. Additionally, in both males and females, 9.3% of all esophageal incident cancer cases occurred in patients below the age of 45 years (Figure 4).

### Gastric cancer incidence over 10 years 2009–2018

**Figure 5.**
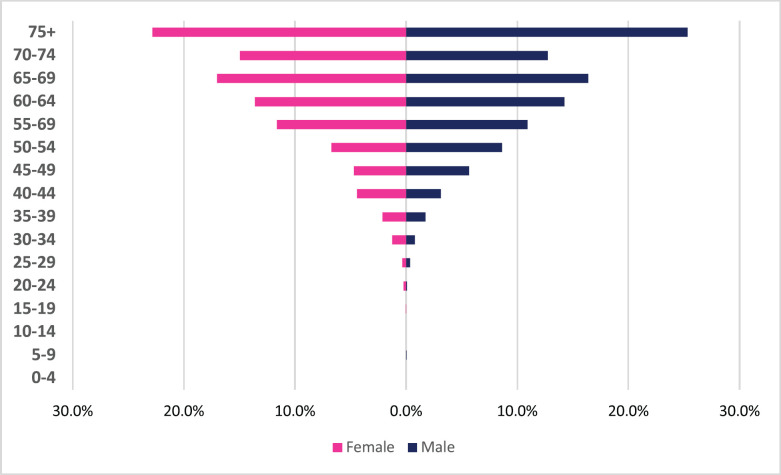
Gastric cancer ASR, per 100,000 females and males from 2009 to 2018.

A total of 2,108 incident gastric cancer cases were diagnosed between 2009 and 2018 with 1,035 male and 1,073 female cases. Gastric cancers accounted for 3.34% of all incident cancer cases in the 10-year period. Among males, gastric cancer accounted for 3.88% of all incident cancer cases while in females it accounted for 2.94% of all cancers. Gastric cancer represented 19.4% of all GI incident cancer cases in Zimbabwe (Supplementary Figure 1a and b). Over the 10-year period, gastric cancer ASR increased in males by 279% from 1.4 per 100,000 to 4.5 per 100,000 in 10 years, and in females increased by 230% from 1 per 100,000 to 3.7 per 100,000 with a male-to-female ratio of 1: 1.2 in 2018. The average annual increase in gastric cancer ASR was 16% in males and 18% in females (Figure 3a and b). Gastric cancer age sub-population analysis showed that 75% of all incident gastric cancer cases were diagnosed in people above the age of 75 years. Only 8.5% of female and 6.1% of male incident cases were diagnosed in people less than 45 years of age (Figure 5).

### Small intestine cancer incidence over 10 years 2009–2018

**Figure 6.**
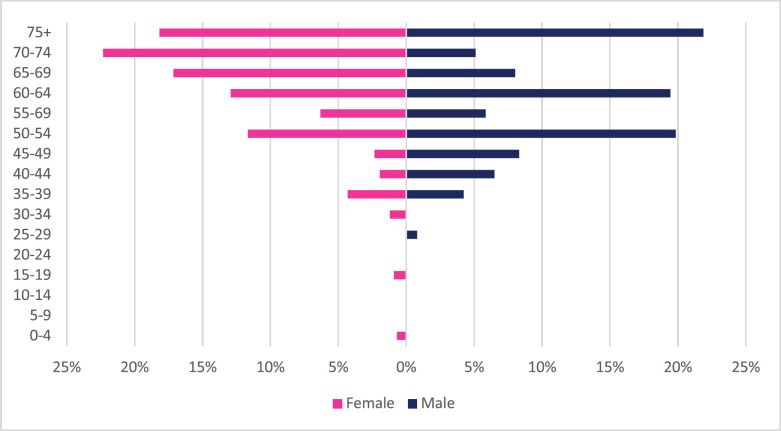
Small intestinal cancer ASR, females and males age subpopulations from 2009 to 2018.

Only 105 incident small intestinal cancer cases were diagnosed between 2009 and 2018 with 54 male and 51 female cases. Small intestinal cancers accounted for 0.17% of all incident cancer cases in the 10-year period. Among males, small intestinal cancers accounted for 0.2% of all incident cancer cases while in females it accounted for 0.1% of all cancers. Small intestinal cancers represented 0.97% of all GI incident cancer cases in Zimbabwe (Supplementary Figure 1a and b). Small intestinal cancer ASR increased in males from 0.1 per 100,000 to 0.3 per 100,000 and in females increased from 0.1 per 100,000 to 0.2 per 100,000 over the 10-year study period. This represented an increase of 114% in females versus 272% in males. The ratio of females to males was 1: 1.5 in 2018. Small intestinal cancer age sub-population analysis showed that 75% of all incident small intestinal cancer cases were diagnosed in people above the age of 50 years. Only 9.1% of females and 11.5% of male incident cases were diagnosed in people less than 45 years of age (Figure 6).

### Colon cancer incidence over 10 years 2009–2018

**Figure 7.**
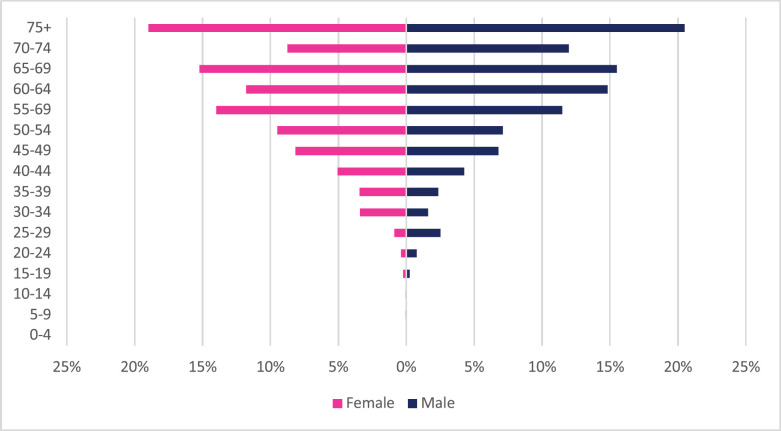
Colon cancer ASR, females and males age subpopulations from 2009 to 2018.

About 1,385 cases of colon cancer were diagnosed with males accounting for 54% of cases. Colon cancer represented 2.19% of all incident cancer cases in Zimbabwe over the 10-year period. Among males, colon cancer accounted for 2.82% of all incident cancer cases while in females it accounted for 1.73% of all cancers. Overall, colon cancers represented 12.7% of all GI incident cancer cases in Zimbabwe (Supplementary Figure 1a and b). Colon cancer ASR increased in males from 1.4 per 100,000 to 3.3 per 100,000 and in females increased from 1.1 per 100,000 to 1.8 per 100,000 over the 10-year study period. The female-to-male ratio in 2018 was 1: 1.8 from an initial 1: 1.3 in 2009. The observed increase in ASR was higher in males compared to females (males 136% versus females 61%). The average annual increase in colon cancer over the 10-year period was 11.47% for males and 8.64% for females (Figure 3a and b). Colon cancer age sub-population analysis showed that 75% of all incident colon cancer cases in females were over the age of 50 years and in males over the age of 55 years. Patients diagnosed below the age of 45 years in females and males represented 13.6% and 11.8%, respectively (Figure 7).

### Rectal cancer incidence over 10 years 2009–2018

**Figure 8.**
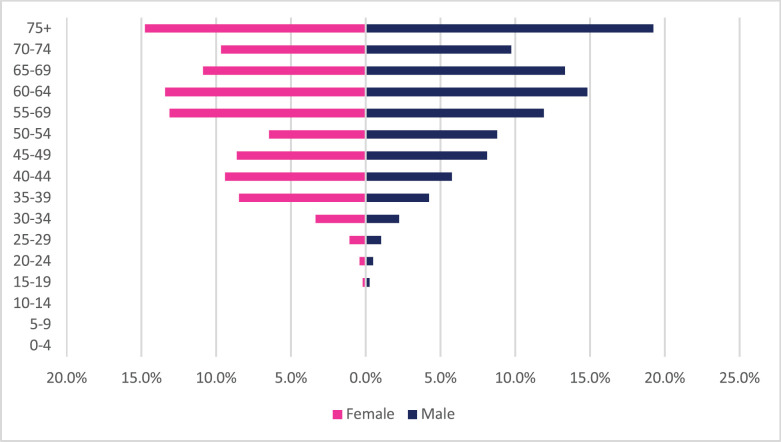
Rectal cancer ASR, female and male age subpopulations from 2009 to 2018.

Rectal cancer incident cases totaled 1,032 with males accounting for 54% of cases. Rectal cancer represented 1.63% of all incident cancer cases in Zimbabwe over the 10-year period. Among males, colon cancer accounted for 2.1% of all incident cancer cases while in females it accounted for 1.3% of all cancers. Rectal cancer diagnosis represented 9.5% of all GI incident cancer cases in Zimbabwe (Supplementary Figure 1a and b). Rectal cancer ASR increased in males from 1.2 per 100,000 to 2.1 per 100,000 and in females increased from 0.6 per 100,000 to 1.5 per 100,000 over the 10-year period. The female-to-male ratio decreased from 1: 2 to 1: 1.4 between 2009 and 2018. The observed increase in ASR of rectal cancers was higher in females compared to males (males 84% versus females 165%) with an average annual increase in colon cancer of 8.3% for males and 13.1% for females (Figure 3a and b). Rectal cancer age sub-population analysis showed that 75% of all incident cancer cases in females were over the age of 55 years and in males over the age of 50 years. 23% and 14.4% of incident cases were diagnosed below the age of 45 years in females and males, respectively (Figure 8).

### Anal cancer incidence over 10 years 2009–2018

**Figure 9.**
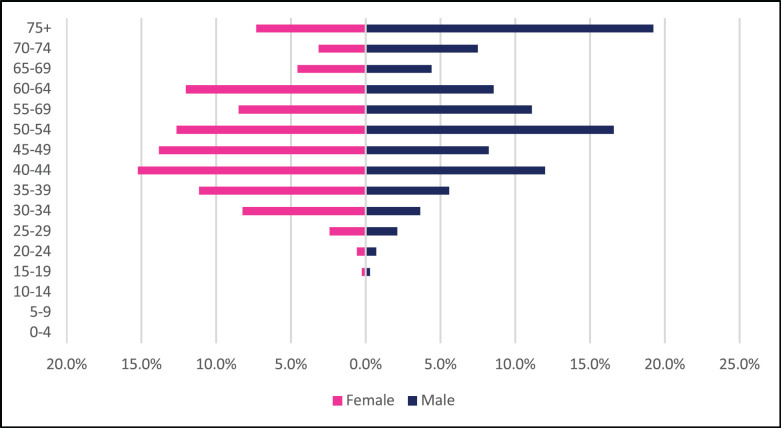
Anal cancer ASR, female and males age subpopulations from 2009 to 2018.

Anal cancer incident cases totaled 335 with males and females accounting for 150 and 185 cases, respectively. Anal cancer accounted for 0.56% of all incident cancer cases in Zimbabwe over the 10-year period. Anal cancer represented 0.56% of all incident cancer cases in males while in females it accounted for 0.5% of all cancers. Overall anal cancer diagnoses represented 3.1% of all GI incident cancer cases in Zimbabwe (Supplementary Figure 1a and b). Anal cancer ASR increased in males from 0.3 per 100,000 to 0.6 per 100,000 in 10 years and in females increased from 0.1 per 100,000 to 0.7 per 100,000. The observed increase in ASR over the 10-year period was significantly higher in females compared to males (males 86.2% versus females 338.06%). The average annual increase in new anal cancer cases over the 10-year period was 11.95% for males and 25.35% for females. This resulted in the male-to-female ratio reducing significantly from 3:1 in 2009 to 1:1 in 2018 (Figure 3a and b). Age sub-population analysis of anal cancers showed that 75% of all incident cancer cases in females are over the age of 40 years and in males over the age of 45 years. Furthermore, in females, 37.9% of incident cases are diagnosed below the age of 45 years compared to 24.3% in males (Figure 9).

### Liver cancer incidence over 10 years 2009–2018

**Figure 10.**
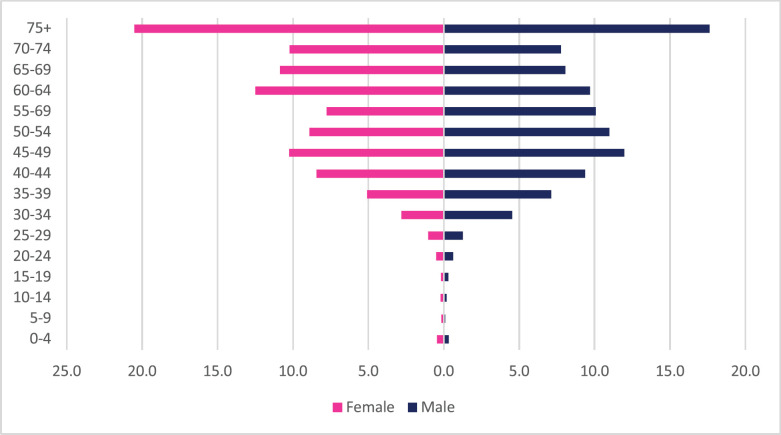
Liver cancer ASR, female and male age-subpopulations from 2009 to 2018.

A total of 1,993 incident liver cancer cases were diagnosed between 2009 and 2018 with males accounting for 65% of cases. Liver cancers accounted for 3.15% of all incident cancer cases in the 10-year period. Among males, liver cancer accounted for 4.83% of all incident cancer cases while in females it accounted for 1.93% of all cancers. Liver cancer represented 18.4% of all GI incident cancer cases in Zimbabwe (Supplementary Figure 1a and b). Liver cancer ASR increased in males from 2.1 per 100,000 to 4.7 per 100,000 and in females increased from 0.9 per 100,000 to 2.3 per 100,000 over the 10-year period. The female-to-male incidence ratio decreased from 1: 2.3 in 2009 to 1: 2 in 2018. The percentage increase in ASR for liver cancer was 124% in males and 151% in females with an average annual increase of 17% and 13%, respectively (Figure 3a and b). Liver cancer age sub-population analysis showed that 18.9% of females and 23.8% of male incident cases were diagnosed in people less than 45 years of age (Figure 10).

### Gall bladder cancer incidence over 10 years 2009–2018

**Figure 11.**
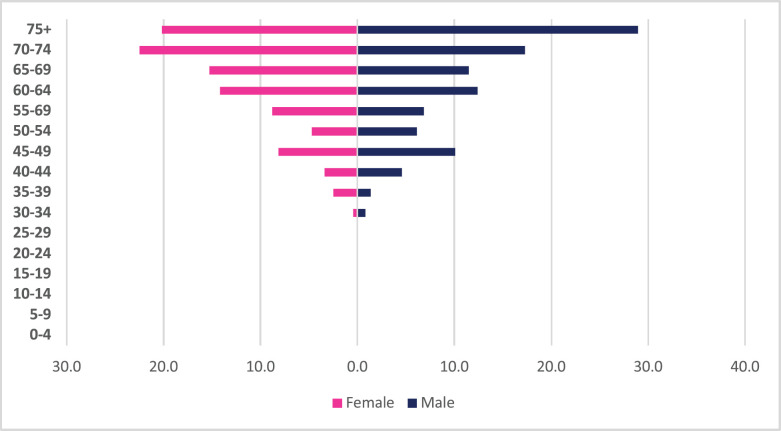
Gall bladder cancer ASR, females and males age-sub-populations 2009–2018.

A total of 255 incident gall bladder cancer cases were diagnosed between 2009 and 2018 with males and females accounting for 118 males and 137 cases, respectively. Gall bladder cancers accounted for 0.4% of all incident cancer cases in the 10-year period. Among males, gall bladder cancer accounted for 0.44% of all incident cancer cases while in females it accounted for 0.38% of all cancers. Gall bladder cancer represented 2.35% of all GI incident cancer cases in Zimbabwe (Supplementary Figure 1a and b). Gall bladder cancer ASR increased in males from 0.14 per 100,000 to 0.42 per 100,000 and in females from 0.1 per 100,000 to 0.4 per 100,000 over the 10 years. The female-to-male ratio was 1: 1 in 2018. The observed increase in ASR was higher in males compared to females (males 310.54% versus females 237.64%). The average annual increase in gall bladder cancer cases was 28.3%% for males and 48.68% for females (Figure 3a and b). Gall bladder cancer age sub-population analysis showed that 6.32% of females and 6.8% of male incident cases were diagnosed in people less than 45 years of age (Figure 11).

### Pancreatic cancer incidence over 10 years 2009–2018

**Figure 12.**
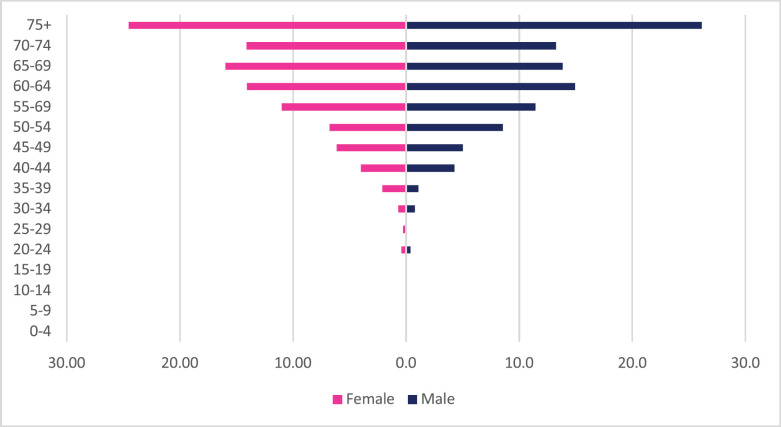
Pancreatic cancer ASR, female and male age-subpopulation 2009–2018.

A total of 663 incident cancer cases were diagnosed between 2009 and 2018 with males and females accounting for 318 and 345 cases, respectively. Pancreatic cancers accounted for 1.05% of all incident cancer cases in the 10-year period. Among males, pancreatic cancer accounted for 1.19% of all incident cancer cases while in females it accounted for 0.94% of all cancers. Pancreatic cancer represented 6.11% of all GI incident cancer cases in Zimbabwe (Supplementary Figure 1a and b). Pancreatic cancer ASR increased in males from 0.28 per 100,000 to 1.7 per 100,000 and in females from 0.7 per 100,000 to 1.2 per 100,000 in 10 years. The increase in ASR over the 10 years was six times higher in males compared to females. The average annual increase in new pancreatic cancer cases over the 10 years period was 24.84% for males and 9.08% for females. The annual increase in ASR was nearly three times higher in males (Figure 3a and b). 1 in 4 pancreatic cancer cases were diagnosed in patients above the age of 75 years. In females and males, 7.5% and 6.8% of all new cancer cases occurred before the age of 45 years, respectively (Figure 12).

## Discussion

### Overall GI cancer trends

Our study showed that Zimbabwe experienced an increase in all GI cancer subtype incidence in both males and females from 2009 to 2018. The increased incidence was higher in males than in females, overall. However, the increase in incidence was observed to be higher in females than males in esophageal, rectal and anal cancers. The overall burden of cancer is increasing worldwide, especially in developing countries. Despite this upward trend, cancer has been given low priority in the research field and in healthcare services in Zimbabwe [31].

### Esophageal cancer

Esophageal cancer had the highest incidence among GI cancers in Zimbabwe. Over the 10 years period, the rate of increase in females was higher than that reported in males. In 1988, the ratio of females to males with esophageal cancer was 1:6.9 and has been decreasing since. In 2009, the female-to-male ratio was 1:3.4 and 1:1 in 2018 [32]. The age-subpopulation comparison between females and males showed a similar pattern which may suggest similar etiological factors. Overall, 1 in 10 new cases were diagnosed in patients below 45 years of age. Age is a top risk factor for esophageal cancer and other factors in Zimbabwe include tobacco smoking, alcohol drinking, low socioeconomic status and working as a miner [33]. Smoking, alcohol consumption and mining work are more prevalent in males [34–36]. Other risk factors include human papilloma virus infection, Barrett’s esophagus, thermal esophageal injury, obesity, micronutrient deficiencies like zinc and selenium, sedentary lifestyle and genetics. Barret’s esophagus has been reported to be rare in a study conducted in six Sub-Saharan African countries that included Zimbabwe and there is postulation that *H. pylori* infection acquired during childhood may offer some protection [37]. Esophageal infection with high-risk HPV subtypes has been described in African patients to be significant; however, there is inconsistent evidence pertaining to HPV genomic integration in esophageal cancer cells [38, 39]. No HPV studies have been done in Zimbabwe esophageal cancer patients. Most patients with esophageal cancer were diagnosed between the ages of 40–74 years of age. Due to the late presentation and delayed diagnosis, the prognosis for most esophageal cancer patients is poor [40]. A definition of the local high-risk population is required to institute public health interventions appropriate for the setting.

### Gastric cancer

Among women, gastric cancer was the second most common GI cancer and it ranked as the third most common in males. One in five new GI cancers in Zimbabwe were gastric cancers. The rate of increase was slightly higher in females compared to males and the male-to-female ratio was 1:1. At diagnosis, 3 in 4 patients were above the age of 75 years. The mortality rates between males and females were similar. Age subpopulation analysis of gastric cancer showed a similar pattern between sexes. The similarity in the incidence of gastric cancer between males and females is a contrast to the global observed pattern of male predominance with a 1:2 ratio [41]. However, in Zimbabwe, the near-equal ratio is a persistent finding. In a review published in 1999, the gastric cancer incidence ASR ratio between females and males was reported as 1: 1.1. An analysis of the risk factor profile and the higher rate of females with gastric cancer is needed. Commonly referenced risk factors such as tobacco smoking, and alcohol consumption will not fully account for the trend observed due to the lower rate of consumption of these substances by Zimbabwe women compared to men [34, 35]. Other forms of possible non-smoking tobacco exposure like curing inhalation and transdermal absorption need to be explored due to the extensive tobacco growing industry in Zimbabwe where women are possibly equally exposed. No studies on this risk factor have been done in Zimbabwe.

### Liver cancer

Liver cancer which is predominantly hepatocellular carcinoma (HCC) was the second most common GI cancer in Zimbabwean men and third in women. The study demonstrated that there is a male predominance with a high rate of annual increase in ASR for both sexes. Our results showed an increasing trend in the incidence of liver cancer over the 10 years. Hepatitis B and C are the main risk factors for liver cancer. Unfortunately, limited data are available on chronic hepatitis in Zimbabwe. It was reported in 2009 in Zimbabwe that 60% of HCC patients had hepatitis B viral infection and 0% were positive for hepatitis C virus [42]. Concerning is the fact that most infected adults are unaware of their status as the disease is predominantly asymptomatic giving viral hepatitis a silent killer status [43]. A significant proportion of newly diagnosed patients are below the age of 45 years with 1 in 4 new male cases. There is faster progression from hepatitis B infection to liver fibrosis and ultimately HCC development in the presence of human immunodeficiency virus co-infection [44]. HIV prevalence in Zimbabwe is approximately 14.1%. Hepatitis B vaccination is the main public health component in the long-term prevention of HCC. Zimbabwe has a reported coverage of +85% and the WHO has set a 90% vaccination target to achieve hepatitis B elimination by 2030 [45]. Routine targeted screening of high-risk populations (hepatitis B and C patients) is needed to enable early diagnosis and intervention.

### Colon cancer

Colon cancer is the fourth most common GI cancer in both sexes. Annually, ASR incidence increased by 11.5% and 8.6% in males and females, respectively. There is a significant proportion of new cases being diagnosed in patients below the age of 45 years. Several factors increase the risk of colon cancer. The prevalence of Lynch syndrome in Zimbabwe is 3.3%. Among the Zimbabwean colorectal cancer patient populations, it has been reported that 18% of patients had genetic risk factors [18]. Changes in diet and lifestyle and increased mining activities, have been reported in the country. A traditional African family diet has been shown to be protective against colon cancer and is associated more with rural primary residence [46].

### Rectal cancer

Rectal cancer is the fifth most common cancer in males and females. The 10-year incidence pattern of the ASR was very similar to that observed in colon cancer. However, this only applied to the ASR. The age subpopulation analysis shows a diverging pattern with 23% and 14.4% of incident cases diagnosed below the age of 45 years in females and males, respectively. This trend of early incidence in females with approximately 1 in 4 new cases being diagnosed in younger patients is unusual. The ZNCR does not provide histological classification in its annual report, leaving a consideration that possibly extensive anal tumors could be included in the rectal tumor category due to the advanced nature of the disease at diagnosis in Zimbabwe. No studies in Zimbabwe have sub-categorised the epidemiology of colon and rectal cancer patterns. There is a similar trend in studies in other Sub-Saharan African countries [47–49]. As the ZNCR improves in details provided, such as histological classification, this will be important data to analyze. The increased rate of females being diagnosed at younger ages requires further investigation.

### Anal cancer

Anal cancer over the past 10 years has undergone a steady increase in ASR. A comparison of males to females also showed that over the time period, the rate of increase in ASR was higher in females at 338% compared to just 86.2% in males. This increase in female anal cancer averaging 25.4% has resulted in a transition to a higher ASR in females compared to males. Anal cancer is an HPV-associated cancer that is globally observed to be increasing as a significant non-AIDS defining cancer. In Zimbabwe, previous studies have shown that HPV was three times more common in females than males with anal cancer [50]. Co-infection with HIV results in faster progression to invasive disease development. The HPV positivity difference between males and females could possibly explain the higher proportion of females being diagnosed at younger ages. Other risk factors identified in the Zimbabwean population for anal cancer include a history of perianal warts, previous cervical intraepithelial neoplasia and reporting more than 10 lifetime sexual partners. HPV vaccination in girls has been introduced in Zimbabwe when?? Using the bivalent HPV vaccine which will provide long-term reduced cancer risk including anal cancer [51].

### Setting and registry limitations

Overall, all GI cancers increased in incidence over the 10-year period we reviewed. It is important to note that the cancer incidence pattern in limited resource settings is a product of an actual increase in disease occurrence and changes in the diagnostic capacity in society. Diagnostic capacity includes access to specimen acquisition such as minimally invasive biopsies, laparoscopic surgery, pathology services and improved imaging capability such as computed tomography (CT) scans, magnetic resonance imaging (MRI) scans and so on.

Moreover, cancer registries in developing countries face many challenges including weak health standards and infrastructures, weak records centralisation, population data analysis and differing health due to developmental, political, social and economic factors. As a result, our study may actually underestimate the incidence rates; however, our endeavour to show the emerging trend shows that greater resources will be needed in NCD care in Zimbabwe in the future.

The ZNCR consistently published annual reports from 2009 to 2018 with improvement in age subpopulation analysis for all cancer types including more detailed data for patients above 75 years of age. Furthermore, mortality data in the latest two reports now has detailed age subpopulation categorised data. Improvement is needed in a primary residence, histological classification, anatomic sub-site description or clinical stage at diagnosis, which will be important to analyse in future studies.

## Conclusion

Zimbabwe experienced an increasing trend in the incidence of all GI cancers over the decade reviewed. The rate of increase in esophageal and gastric cancers in females was particularly high and the male-to-female ratio observed requires further etiological studies. The proportion of young colorectal cancer patients requires both education regarding risk factors and national screening policies that are tailored to the Zimbabwean population characteristics. In resource-limited settings like Zimbabwe, improved data quality and availability are needed to inform effective initiatives and programs aimed at increasing GI cancer awareness, improved cancer prevention and early diagnosis and treatment.

## Conflicts of interest

No conflicts of interest.

## Funding

No funding support for the project.

## Figures and Tables

**Table 1.** Standard population data.

Population	Standard	Reference
**Subgroups**	**population**	**Population weight**
**Age**	**data**	***w_i_***
0–4	88,569.00	0.0886
5–9	86,870.00	0.0869
10–14	85,970.00	0.0860
15–19	84,670.00	0.0847
20–24	82,171.00	0.0822
25–29	79,272.00	0.0793
30–34	76,073.00	0.0761
35–39	71,475.00	0.0715
40–44	65,877.00	0.0659
45–49	60,379.00	0.0604
50–54	53,681.00	0.0537
55–69	45,484.00	0.0455
60–64	37,187.00	0.0372
65–69	29,590.00	0.0296
70–74	22,092.00	0.0210
75+	30,639.00	0.0314
Total	999,999	1.0000

## Supplementary Materials

### Supplementary statistical sheet

#### Calculating age-adjusted rates

- An age-adjusted rate is a weighted average of the age-specific (crude) rates, where the weights are the proportions of persons in the corresponding age groups of a standard population. The potential confounding effect of age is reduced when comparing age-adjusted rates computed using the same standard population.
- The age-adjusted rate for an age group comprised of the ages *x* through *y* is calculated using the following formula [1].
- A crude incidence rate is the number of new cancers of a specific site/type occurring in a specified population during a year, usually expressed as the number of cancers per 100,000 population at risk. It is calculated using the following formula.
- Age-adjusted rates are derived by applying the category-specific incidence and mortality rate (only for 2017 and 2018) of each population GIT subtype to a single standard population (Table 1). This produces ASR that the country would have if it had the same age distribution as the standard population.
- Finally, the age-adjusted rate is calculated by multiplying each crude rate by the appropriate weight and summing the products.
